# Totally Endoscopic Patch Repair for Ruptured Sinus of Valsalva Aneurysm Associated With Ventricular Septal Defect

**DOI:** 10.1093/icvts/ivag136

**Published:** 2026-05-26

**Authors:** Shogo Maeda, Riku Kato, Toshiaki Ito

**Affiliations:** Department of Cardiovascular Surgery, Japanese Red Cross Nagoya Daiichi Hospital, Nagoya 453-8511, Japan; Department of Cardiovascular Surgery, Japanese Red Cross Nagoya Daiichi Hospital, Nagoya 453-8511, Japan; Department of Cardiovascular Surgery, Japanese Red Cross Nagoya Daiichi Hospital, Nagoya 453-8511, Japan

**Keywords:** ruptured sinus of Valsalva aneurysm, ventricular septal defect, totally endoscopic cardiac surgery

## Abstract

Ruptured sinus of Valsalva aneurysm (RSVA) associated with ventricular septal defect (VSD) is a rare but clinically significant condition requiring surgical intervention. We report 2 cases of RSVA with concomitant infundibular VSD successfully treated using a totally endoscopic approach through an aortotomy alone. Both patients were young adults presenting with heart failure symptoms caused by left-to-right shunting. Patch repair of the sinus of Valsalva aneurysm and VSD closure were performed under totally endoscopic visualization. Postoperative echocardiography showed no residual shunt. Both patients were discharged uneventfully. Mild aortic regurgitation was observed but remained stable during mid-term follow-up. Totally endoscopic repair of RSVA associated with VSD could be a feasible option.

## INTRODUCTION

Sinus of Valsalva aneurysm is a rare congenital cardiac anomaly caused by structural weakness of the sinus wall. Ventricular septal defect (VSD) is the most common associated lesion. In ruptured sinus of Valsalva aneurysm (RSVA) with VSD, rupture most frequently occurs from the right coronary sinus into the right ventricle.

RSVA is generally repaired through a standard sternotomy approach. We report 2 cases of RSVA associated with infundibular VSD successfully treated totally endoscopically using an aortic approach alone.

## CASE REPORTS

### Case 1

A man in his 30s from Indonesia presented with exertional dyspnoea. He had a history of congenital heart disease of unknown details. Echocardiography showed preserved left ventricular function (LVEF 63%) and revealed a 5-mm infundibular VSD and an RSVA with a 13-mm neck, with shunt flow from the right coronary sinus into the right ventricle. Contrast-enhanced computed tomography confirmed RSVA without aortic dilatation or coronary abnormalities.

Totally endoscopic repair was performed using a 3D endoscopic system (Karl Storz, Tuttlingen, Germany) equivalent to aortic valve surgery (1). Cardiopulmonary bypass was established via the femoral vessels. After aortic cross-clamping, a C-shaped aortotomy was made. An RSVA arising from the right coronary sinus and a small infundibular VSD beneath the right coronary cusp were identified, with the right aortic annulus located between the 2 defects.

**Figure 1.**
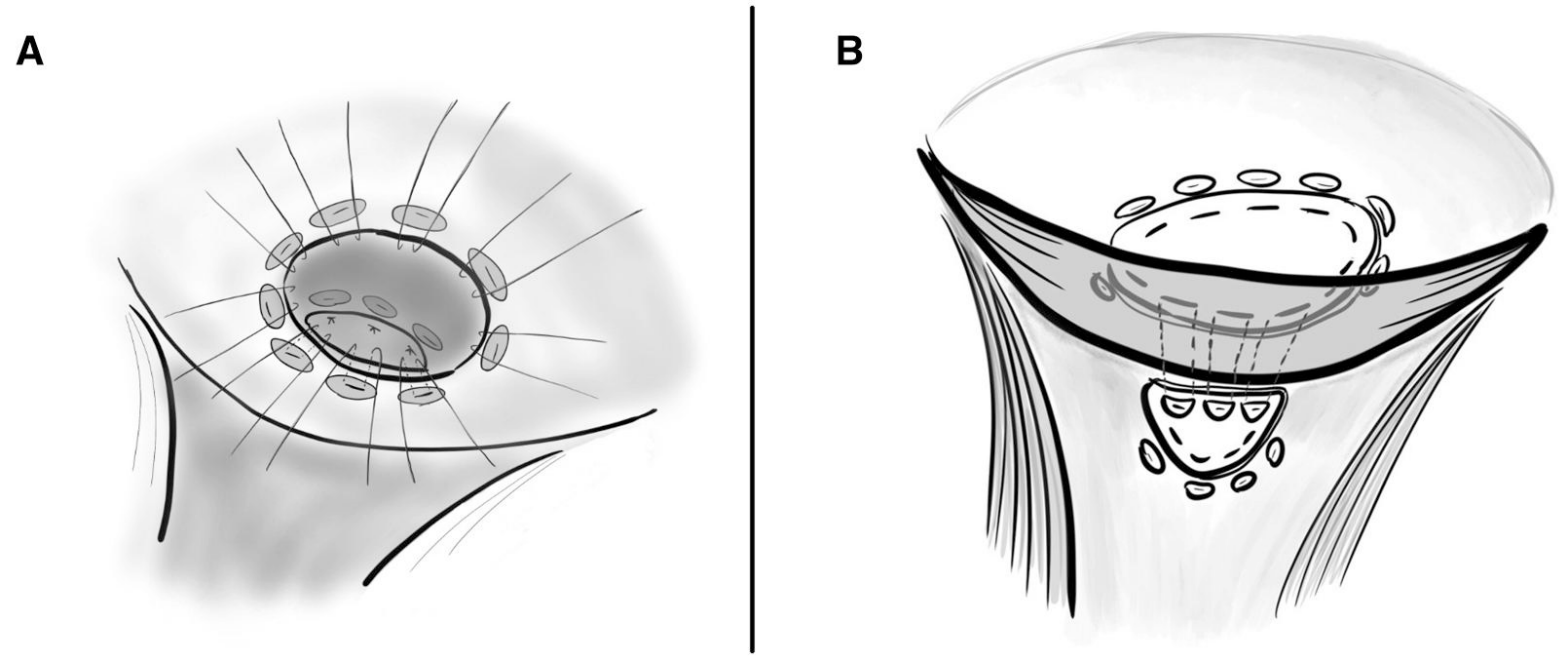
Shema of Surgery in Case 1 (A), and 2 (B)

The aneurysmal wall was resected, leaving the neck intact. Through the RSVA neck, a 0.8-mm expanded polytetrafluoroethylene (ePTFE) patch was secured to the right ventricular side of VSD with mattress sutures. Additional mattress sutures were placed to repair the sinus of Valsalva, and sutures near the annulus were shared with the VSD patch, allowing simultaneous closure (**Figure 1A**, **Video 1**). The aorta was closed in 2 layers.

The postoperative course was uneventful. Mild aortic regurgitation (AR) was observed without residual shunt. The patient is asymptomatic at 4 years of follow-up.

### Case 2

A woman in her 20s from Nepal presented with dyspnoea and chest pain. She had been asymptomatic despite a previously detected heart murmur. Echocardiography revealed an infundibular VSD measuring 10 mm and an RSVA with a 15-mm neck, with shunt flow into the right ventricle. Cardiac catheterization showed a *Q*p/*Q*s of 7.26 with normal coronary arteries.

**Figure 2.**
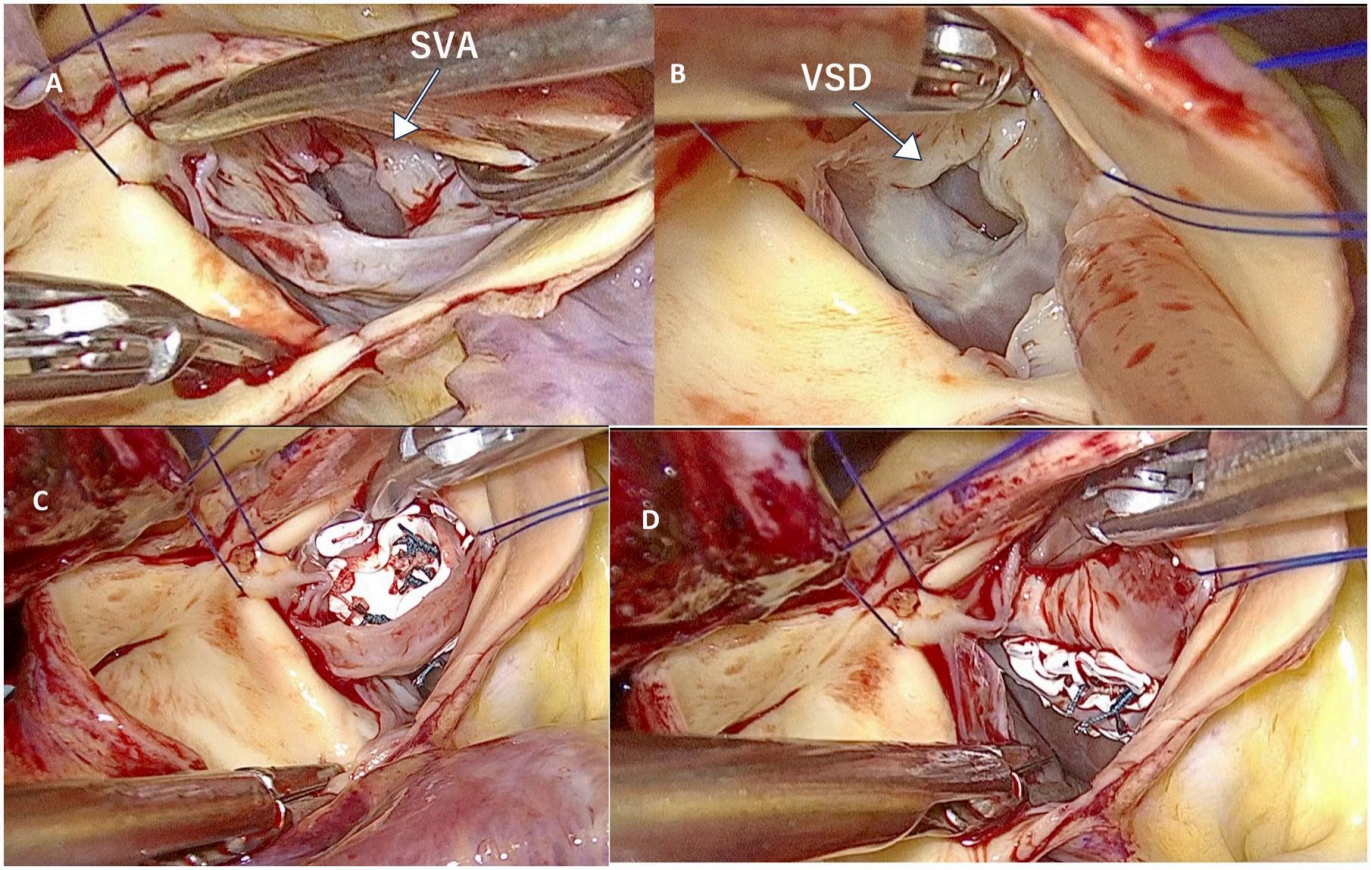
(A) Neck of Sinus of Valsalva, (B) Infundibular Ventricular Septal Defect(VSD), (C) Patch for Valsalva Aneurysm, and (D) Patch for VSD on Left Ventricular Side

Surgical repair was performed using the same setup. Intraoperative findings revealed a ruptured RSVA penetrating the right ventricular outflow tract and a horseshoe-shaped infundibular VSD measuring 8 × 12 mm along the aortic annulus (**Figure 2A and B**). As in Case 1, the annulus of the right coronary cusp was interposed between the RSVA and the VSD.

**Video 1.**
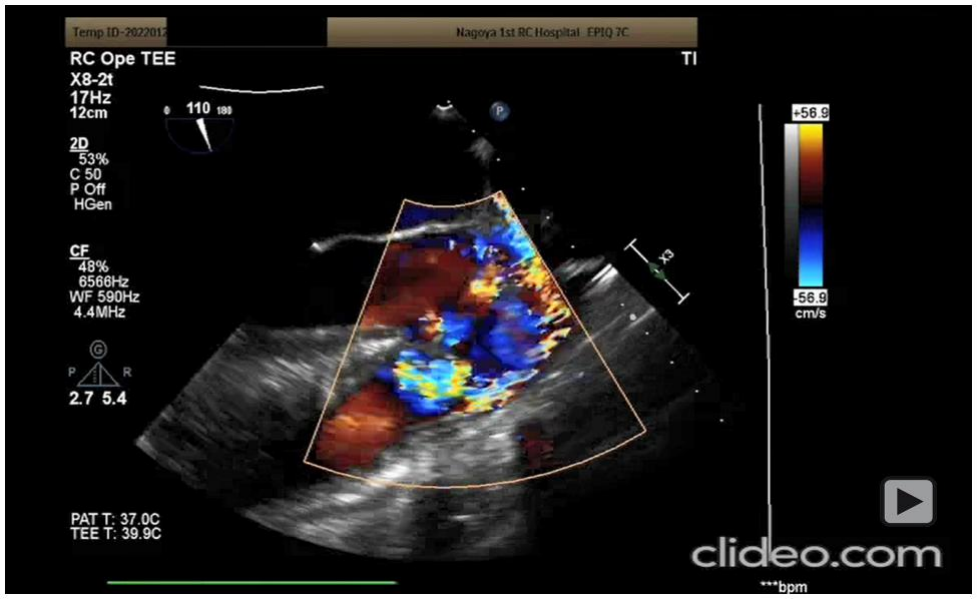
Operative Video and Trans-Oesophageal Echo of Case 1

Because of the larger VSD, patch placement on the right ventricular side through the RSVA neck was technically difficult. Therefore, a 0.8-mm ePTFE patch was secured from the left ventricular side (**Figure 2C and D**). Mattress sutures were passed sequentially through the VSD patch, aortic annulus, and SVA patch to achieve stable closure (**Figure 1B**, **Video 2**).

**Video 2.**
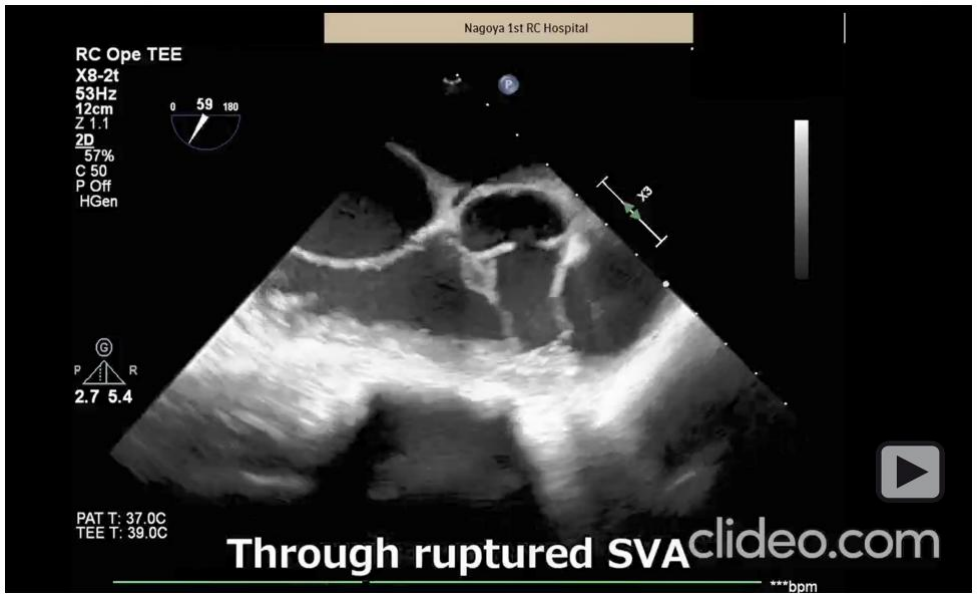
Operative Video and Trans-Oesophageal Echo of Case 2

The postoperative course was uncomplicated without conduction disturbance. The patient was asymptomatic with mild AR at 2-year follow-up.

## DISCUSSION

The aortic approach is widely accepted as the standard surgical strategy for RSVA, allowing direct visualization of the aneurysm neck and facilitating preservation of aortic valve geometry (2). Although additional right ventricular incision may be required in some cases, it increases invasiveness and may impair right ventricular function.

In both cases presented here, totally endoscopic repair using an aortic approach alone was feasible. Three-dimensional endoscopic visualization provided excellent exposure of the spatial relationship among the RSVA, VSD, and aortic annulus, enabling precise anatomical recognition. The choice of patch placement differed according to VSD size: right ventricular-side fixation through the RSVA neck in Case 1 and left ventricular-side fixation in Case 2. Because the conduction system does not course along the margin of infundibular VSDs, left ventricular patch placement can be considered safe.

In both cases, postoperative AR was mild and remained stable; however, long-term follow-up is necessary. Totally endoscopic repair requires advanced experience in endoscopic aortic valve surgery (1) and may not be suitable for all anatomical variations. Careful preoperative assessment and individualized surgical planning are essential.

## Data Availability

All data underlying this article are included within the manuscript. Further details are available from the corresponding author upon reasonable request.
